# Sirolimus monotherapy for Kasabach–Merritt phenomenon in a neonate; Case report

**DOI:** 10.1016/j.ijscr.2024.109497

**Published:** 2024-03-11

**Authors:** Shoji Nakamura, Michio Ozeki, Daichi Hayashi, Shiho Yasue, Saori Endo, Hidenori Ohnishi

**Affiliations:** Department of Pediatrics, Gifu University Graduate School of Medicine, Gifu 501-1194, Japan

**Keywords:** Disseminated intravascular coagulation, Kasabach–Meritt phenomenon, Kaposiform hemangioendothelioma, Vascular tumor, Case report

## Abstract

**Introduction and importance:**

The Kasabach-Merritt Phenomenon (KMP), characterized by thrombocytopenia and consumptive coagulopathy due to endothelial cell growth in the infantile vascular tumor kaposiform hemangioendothelioma, presents a therapeutic challenge. This case highlights the novel use of sirolimus in a neonate, an approach less explored in this age group.

**Case presentation:**

A female neonate presented with a right anterior chest mass, progressing to respiratory distress and congestive heart failure. Diagnosed with KMP, she exhibited low platelet count and coagulation abnormalities. Treatment with sirolimus (0.06 mg/day) led to mass reduction, improved bleeding, and a stable tumor after 12 months, without side effects. This case contrasts with existing literature advocating for combination therapy or higher sirolimus concentrations for effective treatment. Yet, our patient achieved favorable outcomes with low-dose monotherapy, suggesting a potentially safer approach in neonates with immature hepatic and renal metabolism.

**Clinical discussion:**

This case demonstrates the efficacy of low-dose sirolimus monotherapy in treating KMP in a neonate, challenging current preferences for combination therapies or higher doses. It emphasizes the need for further research into age-specific treatment protocols in KMP, considering the unique metabolic profiles of neonates and infants.

**Conclusion:**

Sirolimus has demonstrated potential in treating KMP in pediatric patients. While initial results are promising, determining optimal dosages and trough concentrations, especially in neonates and infants, remains essential.

## Highlights

• Kasabach-Merritt Phenomenon (KMP) is a rare condition characterized by thrombocytopenia and coagulopathy.
• KMP is associated with a high mortality and can be managed with corticosteroids or sirolimus.
• Sirolimus has shown promising results in treating KMP, with improvements in symptoms.
• Optimal sirolimus trough concentrations, especially in neonates and infants, require further research.

## 1 Introduction

Kasabach-Merritt Phenomenon (KMP) is characterized by thrombocytopenia and consumptive coagulopathy, and is caused by the abnormal growth of endothelial cells in the infantile vascular tumors kaposiform hemangioendothelioma (KHE) or tufted angioma [1]. Platelets become trapped in these tumors, leading to excessive clot formation and platelet and coagulation factor consumption, resulting in disseminated intravascular coagulation. KMP associated with KHE is extremely rare, occurring in only 0.07/100,000 individuals [1]. KMP results from rapid tumor growth and invasion, compression of vital structures, or severe hemorrhage, and the mortality rate of it ranges from 10 % to 40 %.

KHE therapy can be very challenging, so conservative treatments with corticosteroids, propranolol, and vincristine have been recommended for managing KHE. However, some patients do not respond to these drugs, and may require more comprehensive approaches that include not only medication, but also radiation, resection, and embolization [2]. Prednisone is commonly used as the first-line therapy for KMP, but its side effects are concerning. Recently, many studies have shown that an inhibitor of mammalian target of rapamycin, sirolimus, has high efficacy for treating KHE with KMP [3]. In a systematic review of sirolimus treatment for vascular anomalies, Fleixo et al. reported that 96 % (50/52) of patients with KMP experienced improvement in symptoms at 6 months and KMP resolved without complications, such as unexpected death [4]. Sirolimus is believed to offer better efficacy with fewer side effects than other therapies [1]. However, the evidence of sirolimus treatment for KMP in neonates and infants remains limited.

Herein we present a case of an infant with KHE in the chest who experienced significant improvement in both the tumor and coagulopathy following sirolimus treatment.

This work has been reported in line with the SCARE criteria [5].

## 2 Case report

**Fig. 1:**
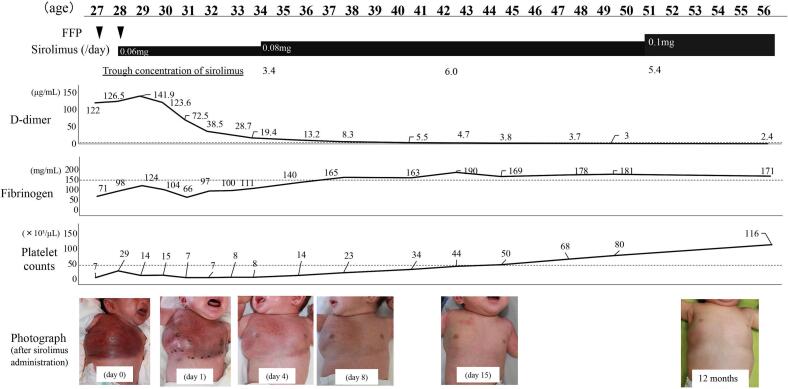
Clinical course of the patient. Treatments, trough concentration of sirolimus, laboratory data (D-dimer, fibrinogen, and platelet count), and clinical photographs are shown. FFP: fresh frozen plasma.

**Fig. 2:**
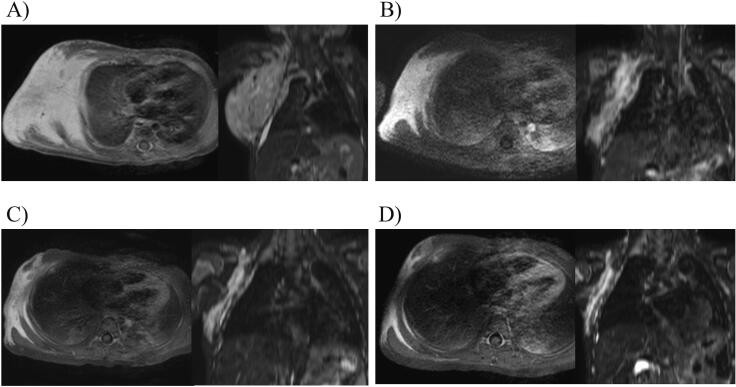
Magnetic resonance imaging of the lesion during treatment. Axial and coronal T2-weighted magnetic resonance imaging of the right chest demonstrates the huge mass infiltrating the right pleura and lung. Before treatment, the tumor compressed the right chest. After treatment, compression from the tumor had reduced. (A) Prior to treatment, (B) 3 months after treatment, (C) 6 months after treatment, and (D) 12 months after treatment.

We report the case of a Japanese female neonate born at 39 weeks and 2 days of gestation, weighing 2964 g at birth. She was referred to our hospital due to a mass on her right anterior chest, initially suspected to be a congenital hemangioma. Shortly after, she exhibited symptoms of respiratory distress and congestive heart failure, necessitating treatment with continuous positive airway pressure and diuretics. She was discharged on day 17; however, the tumor demonstrated growth and internal bleeding, leading to her readmission on day 27. Her right chest showed obvious swelling, with noticeable hardness accompanied by warmth (Fig. 1). Mild respiratory distress was observed caused by compression from the chest tumor. Laboratory investigations revealed mild anemia (hemoglobin: 11.1 g/dL), thrombocytopenia (platelet count: 7000 × 10 [3]/μL) and coagulation abnormalities (fibrinogen concentration: 71 mg/mL; D-dimer concentration: 122 μg/mL), indicative of KMP onset. An MRI revealed an extensive infiltrative tumor in her right chest (Fig. 2A).

With informed consent secured from her parents, we initiated treatment with sirolimus (0.06 mg/day) on day 28, a therapeutic approach endorsed by the review board. We adjusted the sirolimus dosage to achieve trough concentrations between 5 and 15 ng/mL. This treatment led to a reduction in the size of the mass and a marked improvement in the bleeding symptoms and respiratory distress. Notably, D-dimer levels saw a significant drop post-sirolimus administration, while fibrinogen and platelet counts increased. Trough sirolimus levels measured on days 6 and 14 were 3.4 ng/mL and 6.0 ng/mL, respectively, prompting us to incrementally increase the dosage to 0.1 mg/day. By day 28 post-sirolimus initiation, her anemia and thrombocytopenia had fully resolved (hemoglobin: 13.5 g/dL, platelet count: 116 × 10 [3]/μL). The tumor shrunk gradually (Fig. 2B and C). Following coagulopathy normalization, we undertook a tissue biopsy from a skin lesion, which confirmed a KHE diagnosis. One year post-treatment, the tumor remains substantially reduced (Fig. 1, Fig. 2D), and she continues on sirolimus without adverse effects.

This manuscript was prepared following the CARE guidelines (https://www.care-statement.org) [6].

## 3 Discussion

Recently, Ji et al. conducted a randomized clinical trial comparing sirolimus monotherapy (0.8 mg/m^2^/day, target trough concentration, 10–15 ng/mL) with a combined short-term prednisolone regimen (2 mg/kg/day) [3]. The combination therapy group exhibited superior outcomes, including a higher rate of platelet recovery (94.6 % vs. 66.7 %), a reduced transfusion rate (1.4 % vs. 2.5 %), and a decreased risk of recurrence (5.4 % vs. 16.7 %). Importantly, there were no significant differences in adverse effects between the groups. Although our case demonstrated positive results with monotherapy, a combination approach might offer enhanced benefits and reduce recurrence risk. It's evident that more research, particularly studies stratifying treatments based on disease severity, is needed.

Determining the optimal trough concentration of sirolimus for KMP remains a challenge. In our case, the trough concentrations were relatively low, ranging from 3.4 to 6.0 ng/mL. Shan et al. investigated the efficacy of sirolimus across different trough concentration groups (5–10 vs. 10–15 vs. >15 ng/mL), focusing on tumor reduction rate and safety [7]. They found that the group with trough concentrations >10 ng/mL achieved a significantly larger tumor reduction than the 5–10 ng/mL group. However, concentrations exceeding 15 ng/mL did not offer added benefits. Notably, the frequency and severity of adverse effects were consistent across all groups. These results suggest that a trough concentration of 10–15 ng/mL might be optimal. Conversely, Harbers et al. observed that even at a lower sirolimus concentration (4–6 ng/mL), all five patients in their study experienced a full hematological response without adverse effects [8]. Given the immature hepatic and renal metabolism in neonates and infants, which results in reduced drug clearance, a lower target trough concentration might be advisable for this age group [8]. Clearly, more research is essential, especially given the limited experience with sirolimus treatment in neonates.

The majority of cases with KMP are infants, but the safety and tolerance of sirolimus in infants and young children diagnosed with KMP remain underdocumented. Although the common side effects of sirolimus are well-established, only a minority of patients need dose adjustments or temporary cessation due to drug toxicity. Recent research indicates that the maturation of sirolimus clearance continues until the age of 2 years, with clearance being proportional to age [9]. Mizuno et al. proposed estimated age-appropriate starting dosages for sirolimus targeting specific ranges [10]. While the initial dose for our patient was based on the recommended dosing regimen, careful adjustments were made according to the observed trough levels. To establish guidelines on the safe use of sirolimus for pediatric patients, more studies and case accumulations are imperative.

## 4 Conclusion

Sirolimus has demonstrated potential in treating KMP in pediatric patients. While initial results are promising, determining optimal dosages and trough concentrations, especially in neonates and infants, remains essential. This is a single case report, so further research is crucial to establish methods of treatment for its safe and effective use.

## Informed consent

Informed consent was obtained from the guardian. Written informed consent was obtained from the guardian for publication of this case report and accompanying images.

## Consent

Written informed consent was obtained from the patient's parents/legal guardian for publication and any accompanying images. A copy of the written consent is available for review by the Editor-in-Chief of this journal on request.

## Authorship

All authors attest that they meet the current ICMJE criteria for Authorship.

## Ethical approval

Ethical approval for this study was provided by the Ethical Committee National Center for Child Health and Development, Tokyo, Japan on 15 March 2019.

All procedures involving human participants were conducted in accordance with the ethical standards of the committee and the Declaration of Helsinki.

## Funding

The present study was supported in part by a Practical Research Project for Rare/Intractable Diseases (JP22ek0109515) from 10.13039/100009619Japan Agency for Medical Research and Development, AMED.

## Author contribution

S.N., M.O., D.H., S.Y., and S.E. provided medical care for the patient and collected data; S.N. and M.O. wrote the manuscript; H.O. supervised management of the patient. All authors read and approved the final manuscript.

## Guarantor

Michio Ozeki, Department of Pediatrics, Graduate School of Medicine, Gifu University, 1-1 Yanagido, Gifu 501-1194, Japan.

## Research registration number

1. Name of the registry: jRCT
2. Unique identifying number or registration ID: jRCTs031180290
3. Hyperlink to your specific registration (must be publicly accessible and will be

checked): https://jrct.niph.go.jp/latest-detail/jRCTs031180290

## Declaration of competing interest

Michio Ozeki received research funding from Nobelpharma (Tokyo, Japan). The sirolimus tablets were supplied by Nobelpharma. The other authors declare no conflict of interest.
